# Genistein Implications in Radiotherapy: Kill Two Birds with One Stone

**DOI:** 10.3390/molecules30010188

**Published:** 2025-01-05

**Authors:** Xiongxiong Liu, Tong Zheng, Yanyu Bao, Ping Li, Ting Zhao, Yan Liu, Hui Wang, Chao Sun

**Affiliations:** 1 Institute of Modern Physics, Chinese Academy of Sciences, Lanzhou 730000, China; lxx002@impcas.ac.cn (X.L.); zhengtong24@mails.ucas.ac.cn (T.Z.); baoyanyu24@mails.ucas.ac.cn (Y.B.); liping@impcas.ac.cn (P.L.); zhaoting@impcas.ac.cn (T.Z.); 2 Key Laboratory of Heavy Ion Radiation Biology and Medicine, Chinese Academy of Sciences, Lanzhou 730000, China; 3 Key Laboratory of Basic Research on Heavy Ion Radiation Application in Medicine, Lanzhou 730000, China; 4 University of Chinese Academy of Sciences, Beijing 100049, China; 5 School of Medical Imaging, Binzhou Medical University, Yantai 264003, China; liuericyan@hotmail.com

**Keywords:** genistein, radiotherapy, radioprotection, radiosensitization, cancer treatment

## Abstract

More than 70% of cancer patients receive radiotherapy during their treatment, with consequent various side effects on normal cells due to high ionizing radiation doses despite tumor shrinkage. To date, many radioprotectors and radiosensitizers have been investigated in preclinical studies, but their use has been hampered by the high toxicity to normal cells or poor tumor radiosensitization effects. Genistein is a naturally occurring isoflavone found in soy products. It selectively sensitizes tumor cells to radiation while protecting normal cells from radiation-induced damage, thus improving the efficacy of radiotherapy and consequent therapeutic outcomes while reducing adverse effects. Genistein protects normal cells by its potent antioxidant effect that reduces oxidative stress and mitigates radiation-induced apoptosis and inflammation. Conversely, genistein increases the radiosensitivity of tumor cells through specific mechanisms such as the inhibition of DNA repair, the arrest of the cell cycle in the G_2_/M phase, the generation of reactive oxygen species (ROS), and the modulation of apoptosis. These effects increase the cytotoxicity of radiation. Preclinical studies demonstrated genistein efficacy in various cancer models, such as breast, prostate, and lung cancer. Despite limited clinical studies, the existing evidence supports the potential of genistein in improving the therapeutic effect of radiotherapy. Future research should focus on dosage optimization and administration, the exploration of combination therapies, and long-term clinical trials to establish genistein benefits in clinical settings. Hence, the unique ability of genistein to improve the radiosensitivity of tumor cells while protecting normal cells could be a promising strategy to improve the efficacy and safety of radiotherapy.

## 1. Introduction

Cancer incidence has increased worldwide due to the aging global population and changes in lifestyle factors (including unhealthy diets, lack of exercise, smoking habits, and excessive alcohol consumption). According to the World Health Organization (WHO), cancer is one of the leading causes of death worldwide. Research for strategies to enhance the destruction of tumor cells and improve the therapeutic effect while reducing side effects on normal tissues has been underway for some time and is still under investigation. Radiotherapy is one of the several treatments used to cure cancer, which primarily uses X-rays, gamma rays, or other ionizing radiations to target tumor cells [1]. However, the radioresistance of tumor cells and radiation damage to healthy tissues around the tumor are the main obstacles to this treatment. Several radioprotectors and radiosensitizers have been tested in vitro and in vivo to reduce the toxicity in normal tissues while reducing tumor resistance.

Damage to healthy cells can induce acute [2,3] and chronic side effects [4,5,6], affecting the patient’s quality of life. Patient outcomes and quality of life during and post-treatment can be improved by understanding the mechanisms regulating these effects and employing strategies to mitigate them. The Food and Drug Administration (FDA) approved amifostine as a radioprotector in clinical indications [7]. However, no strategy has solved the toxicity problems, with frequent and severe nausea and vomiting. To date, the FDA has not approved new chemicals as radioprotectors for acute radiation syndrome [8]. All FDA-approved radiation countermeasures are classified as radiomitigators, minimizing toxicity post-radiation. For instance, simvastatin reduces cardiac dysfunction and capsular fibrosis [9]. Rapamycin (a specific mTOR inhibitor) mitigates radiation-induced pulmonary fibrosis while protecting hematopoietic cells and the liver [10]. Metformin targets endogenous ROS production inside cells and improves DNA repair capacity [11]. Perindopril increases bone marrow cellularity, and the hematopoietic progenitors CFU-GM, BFU-E, and CFU-Meg mitigate the hematopoietic toxicity [12]. Despite all this progress, an ideal radioprotector has not been identified.

Tumor radioresistance is a major challenge in radiotherapy, which includes intrinsic radioresistance of a subpopulation in the tumor and acquired radioresistance during radiotherapy. Cancer cells increase their radioresistance through various mechanisms, including enhanced DNA repair capabilities [13,14], altered cell cycle dynamics [15], and modulation of the tumor microenvironment [16]. Although many studies have been performed on tumor radioresistance mechanisms [17], there is no effective way to address this problem in clinical practice. Further research should focus on finding agents with radioprotective and radiosensitive properties for radiotherapy applications. Fortunately, natural compounds are safer compared to synthetic chemical compounds due to their high safety level.

Several natural compounds have been studied for their potential anticancer effects. For instance, the in vitro and in vivo anticancer activities of basil (*Ocimum* spp.) have been examined, highlighting its potential application in cancer therapy [18]. Genistein is a prominent isoflavone identified in soybeans with multifaceted roles in cancer biology. It is known for its antioxidant properties and ability to modulate various signaling pathways. Its anticancer effects consist of suppressing cell proliferation, inducing apoptosis, and limiting metastasis [19,20]. Its potential synergy with radiotherapy offers a promising avenue to improve treatment outcomes and reduce side effects. This effectively allows clinicians to “kill two birds with one stone”. Clinical trials are performed using genistein alone or as a co-treatment with other compounds. A phase II clinical study has been performed to explore the ability of genistein to prevent or reduce heart disease and diabetes risk among men receiving androgen deprivation therapy for prostate cancer (ClinicalTrials. gov ID: NCT02766478). Another phase II trial is assessing the therapeutic effect and molecular mechanism of genistein on prostate cancer (ClinicalTrials.gov ID NCT01126879). Other phase II clinical trials have been performed to study the therapeutic effects of genistein combined with other chemotherapeutics on solid tumors (ClinicalTrials.gov ID: NCT00244933 and NCT01628471). Recently, some studies have demonstrated that genistein has radiosensitive effects on tumor cells and radioprotective effects on normal cells. This review will elucidate the mechanisms through which genistein functions as both a radioprotector and a radiosensitizer, highlighting its potential applications in cancer radiotherapy.

Genistein has radiosensitive effects on tumor cells and radioprotective effects on normal cells, thus being potentially used as a complementary agent in radiotherapy regimens by enhancing the cytotoxic effects of radiation on tumor cells while protecting normal tissues. This review clarifies the mechanisms used by genistein to exert radioprotection and radiosensitizing with future applications in cancer radiotherapy, examining its biological mechanisms, clinical relevance, and potential impact on treatment efficacy to provide insights into the therapeutic potential of this natural compound while promoting future clinical strategies against cancer.

## 2. Radiotherapy as a Double-Edged Sword in Tumor Treatment

Radiotherapy targets and destroys cancer cells with high precision, but its application presents significant challenges and risks, making it a double-edged sword. Radiotherapy precisely targets tumor cells, delivering high radiation doses to kill them or inhibit their growth. It is particularly effective in localized tumors and is used as a primary treatment modality or combined with surgery and chemotherapy. Its use in palliative care helps to alleviate pain and other symptoms caused by the tumor, such as obstruction or bleeding, improving the quality of life in advanced cancer patients [21]. Despite its precision, radiotherapy can affect surrounding healthy tissues. Radiotherapy induces damage to cells by causing direct breaks within the DNA strands [22], producing ROS and damaging cellular components, including DNA, proteins, and lipids [23], thus triggering an inflammatory response [24]. Nevertheless, radiotherapy aims to maximize the killing of cancer cells while minimizing injury to nearby healthy tissues. Therefore, further promising adjuvants for radiotherapy should increase the radiosensitivity of cancer cells and decrease the effects on healthy cells. Natural compounds have relatively low cytotoxicity effects, with radiotherapeutic potential. Therefore, identifying these agents may enhance the effects of cancer cell death in response to radiotherapy at one point and protect healthy tissues against radiation-induced damage at another.

### 2.1. Genistein as a Radioprotector

**Figure 1:**
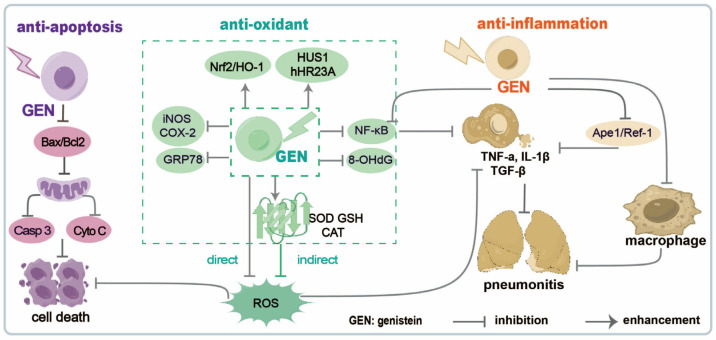
Mechanisms of radioprotection exerted by genistein.

Genistein has gained significant interest for its potential protective effects against radiotherapy-induced normal tissue damage. The protective mechanisms of genistein were investigated in vivo and in vitro. A study by the Michael RL group demonstrated that a single subcutaneous genistein administration at non-toxic doses (100, 200, or 400 mg/kg) 24 h before irradiation significantly increased the 30-day survival rates of mice [25]. The survival rate of mice depends on radioprotection against acute myelotoxicity while promoting the proliferation of the hematopoietic stem cells [26,27] or protection against acute intestinal [28,29], lung [30,31], liver [32], and testicular injuries induced by radiation [33]. Jackson IL. et al. showed that genistein increases the radiotherapeutic effects on prostate cancer without decreasing tumor radiosensitivity and preventing radiation-induced erectile dysfunction [34]. A clinical trial showed that genistein reduces radiotherapy-induced adverse symptoms in the bladder and rectum of prostate cancer patients [35]. Genistein mitigates several cytotoxic effects of radiation on healthy tissues by inducing antioxidant, antiapoptotic, anti-inflammatory, and anti-fibrotic effects by the modulation of several signaling pathways (Figure 1) that are involved in these processes [36,37,38].

#### 2.1.1. Genistein Reduces Radiation-Induced Apoptosis in Healthy Cells

Radiation-induced apoptosis is one of the main forms of cell death, causing significant damage to healthy tissues surrounding the tumor. Genistein exerts its protective effects by modulating various signaling pathways associated with apoptosis. The mitochondrial pathway is the main route used by radiation to cause apoptosis. Indeed, radiation induces the loss of mitochondrial membrane potential, which in turn causes the release of cytochrome c and the activation of caspases, which are crucial apoptosis mediators. Genistein stabilizes the mitochondrial membrane potential by scavenging radiation-induced ROS and alleviating oxidative stress-induced damage to the mitochondrial membrane. It regulates intracellular Ca^2+^ homeostasis by reducing the influx of extracellular Ca^2+^ and ensuring the normal uptake of mitochondrial Ca^2+^ [39,40]. These effects help protect healthy cells from apoptosis. Moreover, Bcl-2-associated X apoptosis regulator (Bax) protein is involved in the promotion of mitochondria outer membrane permeability by forming pores in the mitochondrial outer membrane [41,42]. Haddad YH et al. demonstrated that genistein reduces Bax and increases Bcl-2 expression, leading to a reduced Bax/Bcl-2 ratio by 50%, subsequently repressing caspase 3 expression and inhibiting the release of cytochrome C from the mitochondria [43]. Kim JS et al. [33] reported that genistein (200 mg/kg body weight) administered by a subcutaneous injection to male C3H/HeN mice 24 h before irradiation (5 Gy) protected the germ cells from testicular dysfunction induced by gamma irradiation through an antiapoptotic effect. A low genistein concentration (1.5 µM) protects L-02 cells against radiation damage by inhibiting apoptosis, downregulating GRP78 and upregulating HERP, HUS1, and hHR23A [44]. The ability of genistein to inhibit radiation-induced apoptosis in healthy cells is a crucial mechanism that protects them from harmful side effects.

#### 2.1.2. Genistein Protects Healthy Cells from Radiation-Induced Oxidative Damage

Genistein possesses potent antioxidant properties that are critical in mitigating radiation-induced damage to healthy tissues by two distinct effects: the direct and indirect ones. The direct effect consists of scavenging free radicals, involving ROS and nitrogen species [45,46], while the indirect antioxidant effect consists of the decrease in the expression of ROS-producing enzymes, including SOD1 and HO-1 [47]. Genistein directly scavenges ROS, neutralizing them before any damage [33,48], downregulates ROS induced by irradiation, alleviates radiation-induced pneumonitis [49], and upregulates the expression and activity of endogenous antioxidant enzymes [50,51], thus being vital in detoxifying ROS and protecting cells from oxidative stress. Genistein helps to maintain redox balance and reduces the oxidative damage of healthy tissues by enhancing the natural antioxidant defenses during radiotherapy. It has been proposed that some upstream genes, such as the nuclear factor erythroid 2 (NF-E2)-related factor 2 (Nrf2)/heme oxygenase-1 (HO-1) pathway or TGF-β signaling, are involved in regulating antioxidant enzymes. The Nrf2 pathway activation by genistein increases the production of phase II detoxifying enzymes and antioxidant proteins, inducing a robust defense against oxidative damage [52,53,54]. Genistein exerted radioprotective effects by activating Nrf2/HO-1 signaling, subsequently attenuating oxidative stress and apoptosis of healthy lung cells [55]. Another vital antioxidant effect of genistein is inhibiting ROS/NO-producing enzymes [47,56] and hemolysate-induced iNOS and COX-2 in astrocytes at the transcriptional level [57]. These properties may make genistein a potent radiation mitigator by modulating ROS/NO metabolism after radiotherapy. Moreover, the antioxidant properties and protective effects of genistein against radiation-induced damage on healthy tissues were demonstrated in vivo in several animal studies. A study on mice exposed to radiation revealed that genistein administration significantly decreases oxidative stress markers and protects against DNA damage in healthy tissues [36]. Another study revealed that genistein reduces 8-OHdG expression and protects against DNA damage by reducing oxidative stress in the lungs of rats after irradiation [33]. Furthermore, preliminary clinical trials have shown that genistein supplementation can decrease oxidative stress and its associated side effects in cancer patients undergoing radiotherapy. Lower levels of oxidative stress markers were found in patients receiving genistein in a pilot study. They reported fewer side effects, including skin reactions and fatigue, than those not receiving the supplement [58]. Preclinical and early clinical studies support the efficacy of genistein in decreasing oxidative stress and improving patient outcomes during radiotherapy.

All these loads of evidence demonstrated the potent antioxidant properties of genistein in protecting healthy tissues from radiotherapy-induced damage.

#### 2.1.3. Genistein Reduces Radiation-Induced Inflammation and Fibrosis in Healthy Cells

Genistein downregulates the production of proinflammatory cytokines, which are typically increased after radiation exposure. The NF-κB pathway is a critical inflammatory regulator [59], which is activated by radiation, leading to the transcription of different proinflammatory genes. Genistein exerts an inhibitory effect on NF-κB activation, being a potential candidate as an anti-inflammatory agent [57,60,61,62,63]. Genistein exerts its anti-inflammatory properties by suppressing the secretion of proinflammatory cytokines while inhibiting the activation of inflammatory pathways after radiation [64,65,66,67]. Radiation-induced pneumonitis and subsequent pulmonary fibrosis can occur after radiotherapy to the chest, causing respiratory issues and reducing lung function. Genistein treatment decreases the expression of the inflammatory cytokines TNF-a, IL-1β, and TGF-β in healthy lung tissue, providing partial protection against the early effects of lung irradiation while reducing the extent of fibrosis [38]. A similar study demonstrated that genistein reduces radiation-induced inflammatory cytokines by inhibiting the expression of apurinic/apyrimidinic endonuclease 1/redox factor-1 (Ape1/Ref-1) expression, thus attenuating the inflammatory response and pneumonitis [49]. Alveolar macrophages are essential in the upregulation of the inflammatory process after irradiation. The administration of a genistein-enriched diet to mice reduces macrophage accumulation and decreases fibrosis development in their lungs due to irradiation [68,69]. Genistein also mitigates the radiation-induced immune response in mice by reducing leukocyte infiltration in the lungs after the daily administration of soy isoflavones [70]. Thus, genistein effectively mitigates inflammation and subsequent tissue damage. Preclinical and clinical studies support the potential of genistein as an additional therapy to decrease the side effects induced by radiotherapy.

### 2.2. Genistein as a Radiosensitizer

**Figure 2:**
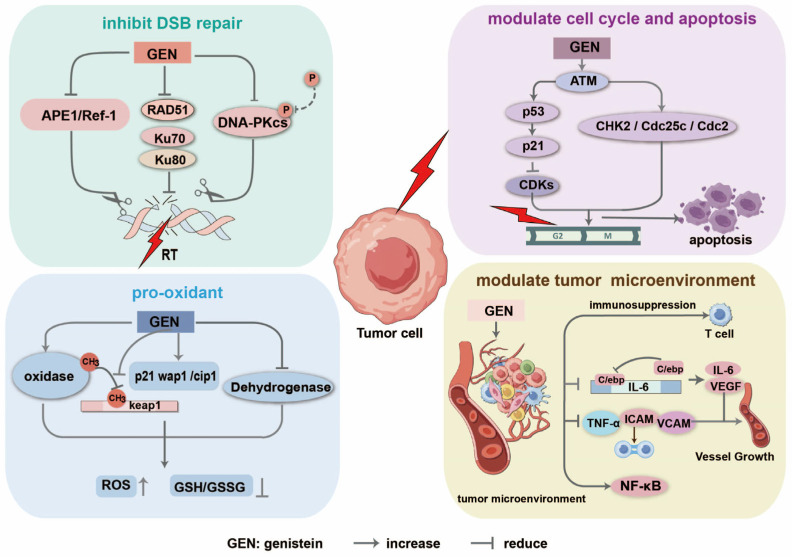
Mechanisms of action of the radiosensitive effect of genistein on cancer cells.

Radioresistance is a significant challenge in radiotherapy, as it decreases its efficacy against certain cancer cells. Tumor heterogeneity is responsible for the different responses to radiation of different tumor subpopulations [17,71]. The increase in tumor radiosensitivity, especially the ability to kill cancer stem cells, is a significant challenge in radiotherapy. Other than the potent radioprotective effects of genistein, some studies have demonstrated its tumor radiotherapeutic effects. It increases radiosensitivity by several mechanisms, such as reducing DNA repair ability, modulating G_2_/M arrest and apoptosis, selectively enhancing radiation-induced oxidative stress, and modulating the tumor microenvironment against tumor cells (Figure 2).

#### 2.2.1. Modulation of DNA Double-Strand Breaks and Repair in Tumor Cells

Radiosensitization agents target DNA damage response, potentially improving the therapeutic effect on tumor cells. Radiation induces various DNA lesions, and DNA DSBs are the most significant. Genistein increases the effect of radiotherapy by inhibiting radiation-induced DSBs while interfering with the repair mechanisms in tumor cells, thereby improving therapeutic outcomes. Two pathways are involved in the repair of DNA DSBs: the non-homologous end joining (NHEJ) and homologous recombination (HR) pathways [72]. The sensor proteins Mre11-Rad50-Nbs (MRN) complex [73] and Ku70/80 [74] are competitively integrated at the end of DSBs in response to it. Our group and others observed that the binding of the Ku heterodimer to DSB ends is faster than that of the MRN complex [75]. These sensor kinases initiate downstream signaling responses and recruit downstream repair factors to complete DSB repair. One study demonstrated the role of NHEJ-specific DNA-PKcs in repairing radiation-induced DSBs in tumor cells [76]. Our previous study demonstrated that DNA-PKcs is involved in HR and NHEJ repair pathways. Genistein physically binds to DNA-PKcs at the 3557–4128 aa site and functionally suppresses DNA-PKcs phosphorylation, repressing NHEJ and delaying the HR repair pathway [22]. It reduces the expression and activity of proteins involved in DNA repair, including DNA-PKcs, Ku70, Ku80 (key players in NHEJ), and Rad51 (a key player in HR), and impairs the ability of tumor cells to repair DNA DSBs efficiently by downregulating these proteins, increasing cell death after radiation exposure [77]. Soy isoflavones, consisting of 83.3% genistein, inhibit DNA repair by inhibiting the APE1/Ref-1 pathway, which is involved in DNA repair, causing an increased radiation-induced killing of NSCLC cells [78]. The inhibition of radiation-induced DSBs by genistein and its interference with DNA repair mechanisms in tumor cells represent a promising strategy to improve the efficacy of radiotherapy by impairing key DNA repair pathways, decreasing repair protein levels, enhancing DNA damage, and finally sensitizing tumor cells to radiation.

#### 2.2.2. Modulation of Cell Cycle and Apoptosis in Tumor Cells

Genistein induces cell cycle arrest specifically in the G_2_/M phase [79,80], which represents a significant target to improve cancer radiotherapy [81,82]. This arrest leads to the accumulation of DNA damage and prevents the mitosis of cells with damaged DNA. Genistein activates the ataxia-telangiectasia mutated (ATM) kinase that is involved in the cellular response to DNA damage. ATM activation leads to the stabilization and activation of the p53 tumor suppressor protein, which in turn triggers the expression of the cyclin-dependent kinase inhibitor p21. This inhibits the activity of cyclin-dependent kinases (CDKs) necessary in the transition from G_2_ to M phase, leading to G_2_/M arrest [83,84]. Our previous study suggested that genistein induces G_2_/M arrest by activating the ATM/Chk2/Cdc25C/Cdc2 checkpoint pathway and by enhancing the radiosensitivity of breast cancer cells through a mitochondria-mediated apoptosis pathway [85]. Moreover, several other studies showed that genistein improves the radiosensitivity of different cancer cells by inducing G_2_/M arrest and promoting cell apoptosis using multiple mechanisms [63,86,87]. Thus, genistein sensitizes tumor cells to radiotherapy by regulating cell cycle checkpoints, activating apoptotic pathways by modulating the balance of pro- and antiapoptotic proteins, thus improving the effect of radiotherapy.

#### 2.2.3. Selective Increase in Radiation-Induced Oxidative Stress in Tumor Cells

Flavonoids exert pro-oxidant effects on tumor cells and antioxidant effects in healthy cells. Genistein activates pro-oxidant enzymes, including NADPH oxidase, in tumor cells. This activation increases ROS production, enhancing oxidative stress [88]. Our previous study demonstrated that genistein increases ROS levels by inhibiting the methylation of the keap1 gene promoter region in NSCLC cells. In contrast, oxidative stress is reduced by increasing the expression of Nrf2, GSH, and HO-1 in MRC-5 cells, selectively improving the radiosensitizing effect in NSCLC A549 cells [55]. Genistein suppresses the expression of cytoplasmic nicotinamide adenine dinucleotide phosphate (NADP)-dependent isocitrate dehydrogenase, which increases the levels of intracellular ROS by upregulating p21wap1/cip1 and decreasing GSH/GSSG induced by radiation. Genistein and γ-irradiation synergistically induce cell death in leukemia cells by exerting a pro-oxidant activity, although they have an antioxidant effect on healthy human lymphocytes, selectively enhancing the radiosensitivity of leukemia cells [89]. Genistein increases radiation-induced oxidative stress and apoptosis in lung cancer cells with minimal effects on healthy lung cells [55]. The ability of genistein to selectively improve radiation-induced oxidative stress in tumor cells is a promising effect to increase the efficacy of radiotherapy. Genistein sensitizes tumor cells to radiation by increasing the production of ROS that selectively accumulate in these cells, inhibiting antioxidant defenses, and activating pro-oxidant enzymes, all factors increasing cell death.

#### 2.2.4. Modulation of Inflammation and Tumor Microenvironment Against Tumor

A malignant tumor is composed of tumor cells and a wide range of normal host tissues, including stroma, vasculature, and immune infiltration. Tumor cells corrupt the host tissues to use them to support their growth, therapy resistance, and evade immune defense. Thus, therapies that disrupt the supportive tumor microenvironment or inhibit stromal cell survival signals make cancer cells more sensitive to radiation [90]. Genistein modulates the synthesis of inflammatory cytokines, which are mediators of the immune response to radiation by increasing the inflammatory response in the tumor through the elevated expression of proinflammatory cytokines, such as TNF-α, IL-6, and IL-1β, rendering it more susceptible to radiation [49,91]. Genistein inhibits C/EBP β interaction with the IL-6 promoter, disturbing the angiogenic microenvironment, thus reducing VEGF and IL-6 expression and inhibiting the proliferation of EGFRvIII-positive glioblastoma [92]. Genistein administration in colon cancer rats inhibits the expressions of the colonic stem cell marker proteins CD133, CD44, and β-catenin, thus suppressing colon cancer progression [93]. Radiotherapy has significant effects on the tumor microenvironment and induces proinflammatory effects and immune suppressive effects that can either promote or inhibit antitumor immunity [94]. Moreover, genistein regulates the gene expression of several proinflammatory molecules, such as NF-κB, activator protein-1, intercellular adhesion molecule-1 (ICAM), vascular cell adhesion molecule-1 (VCAM), and E-selectins [95,96]. Thus, genistein increases radiation-induced inflammation and modifies the tumor microenvironment to enhance the therapeutic effects of cancer treatment.

#### 2.2.5. Targeting Other Signaling Pathways

Genistein is a well-known estrogen receptor (ER) agonist. Hermann RM et al. demonstrated that 10 μM genistein increases low-dose hyper-radiosensitivity in ER-α- and ER-β-positive but not negative prostate cancer cells [97]. p53 protein is the most critical antitumor barrier. Our previous study revealed that genistein does not alter the p53 expression in MCF-7 (p53 wild-type) and MDA-MB-231 (p53 mutant) breast cancer cells, and the radiosensitization of genistein is achieved through a p73-mediated manner [85]. However, another study revealed that genistein induces cell death in a cell line with wild-type p53 in a different way than in the cells with the mutant p53. Nevertheless, genistein significantly suppresses the radiation-induced activation of p42/p44 and AKT/PKB survival signals, thus enhancing radiosensitivity in human esophageal squamous cell carcinoma cell lines [98].

### 2.3. Genistein Selectively Enhances the Radiosensitivity of Tumor Cells and the Radioprotection of Normal Cells

**Figure 3:**
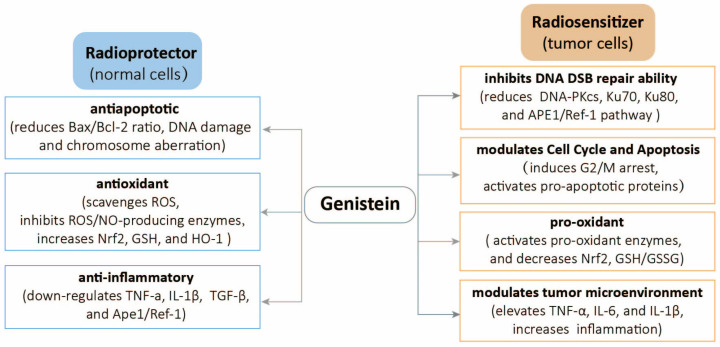
The distinction mechanisms of genistein in healthy and tumor cells.

Genistein acts through distinct mechanisms in healthy and tumor cells (Figure 3); it selectively improves the radiosensitivity of tumor cells and protects healthy cells from radiation damage. This selective action is crucial for improving the effect of radiotherapy, providing better cancer control with fewer side effects. Das A et al. reported that genistein selectively phosphorylates p38 MAPK by activating the redox-sensitive JNK1 pathway, thus inducing apoptosis in human glioblastoma T98G and U87MG cells without impacting healthy astrocytes [99]. Soy isoflavones radiosensitize lung cancer to radiation while reducing hemorrhages, inflammation, and fibrosis induced by radiation in healthy lung tissues [36]. Our previous study demonstrated that genistein selectively sensitizes NSCLC A549 cells but not healthy lung MRC-5 fibroblasts to radiation [50]. The daily nano-genistein doses of 200–400 mg/kg significantly sensitize mouse lung tumors to radiation and protect healthy lung tissues by reducing pulmonary congestion, inflammation, and collagen deposition [100]. A clinical trial demonstrated that prostate cancer patients receiving soy isoflavones during and after radiotherapy have a higher reduction in prostate-specific antigen with a reduced incidence of urinary, gastrointestinal, and erectile dysfunction than those receiving placebo [35]. These results suggest that genistein administered in conjunction with radiation therapy selectively increases the tumor-killing effect and mitigates radiation injury to the adjacent healthy tissues. This dual role of genistein represents a promising strategy to improve the effect of radiotherapy by inhibiting DNA repair and enhancing oxidative stress and apoptosis in tumor cells while providing antioxidant and anti-inflammatory protection to healthy tissues. The evidence from preclinical studies supports this potential, although additional clinical research is needed to optimize the use and confirm the benefits of genistein in clinical settings. This approach may provide more effective cancer treatments with reduced side effects, improving the overall patient outcomes.

## 3. Conclusions and Perspectives

The selective elimination of cancer cells without harming healthy cells is a fundamental challenge in cancer therapy. Soy isoflavones, which consist of genistein, daidzein, and glycitein, possess important properties that selectively enhance the radiosensitivity of tumor cells and protect normal cells from the effects of radiation [101,102]. Moreover, some other naturally occurring substances act as radiosensitizers on cancer cells and radioprotectors on normal cells. Both curcumin and resveratrol are well-known plant-derived polyphenols. They have antioxidant/anti-inflammatory effects on normal cells and cause pro-oxidant/G_2_-M arrest in cancer cells [103,104,105,106]. The combination of Astragalus mongholicus Bunge and its small molecule ononin increases the radiosensitivity of lung cancer while simultaneously protecting liver function from radiation damage in C57BL/6 mouse models [107]. Nevertheless, the evidence from clinical trials is very limited.

Genistein is a low-toxic agent with versatile pharmacological activities, including anti-inflammatory [108], antioxidant [109], antiangiogenic [110], anticancer [111], and antiproliferative effects [112]. The role of genistein in radiotherapy has gained considerable attention due to its dual effect of increasing the radiosensitivity of tumor cells and simultaneously protecting healthy tissues from radiation-induced damage.

This unique property makes genistein an attractive adjunct in cancer treatment, potentially improving therapeutic outcomes and decreasing adverse effects. Genistein exerts radioprotective effects through multiple pathways, such as antioxidant, antiapoptotic, and anti-fibrotic activities. Moreover, it has strong inhibitory activities against radiation-induced inflammatory damage on healthy tissues by inhibiting several signaling pathways and molecules, including NF-κB, proinflammatory cytokines, and ROS [31,38,57,58,59,60,61,62]. This anti-inflammatory effect prevents long-term radiation-induced tissue fibrosis and other chronic side effects.

Genistein protects healthy cells while increasing the radiosensitivity of tumor cells through various mechanisms. It effectively inhibits the DSB repair ability in tumor cells. The most relevant mechanism of genistein is that it physically binds to DNA-PKcs at the 3557–4128 aa site and functionally inhibits the DSB repair pathway [22]. Genistein reduces the expression of repair proteins, such as DNA-PKcs, Ku70, Ku80, and RAD51. Moreover, it induces cell cycle arrest at the G_2_/M phase where cells remain sensitive to radiation. This arrest leads to the accumulation of unrepaired DNA damage, enhancing the cytotoxic effects of radiation on tumor cells. Genistein promotes apoptosis in tumor cells by activating both intrinsic (mitochondrial) and extrinsic (death receptor) apoptotic pathways. Furthermore, it increases ROS production in tumor cells when combined with radiation, which in turn induces oxidative stress and DNA damage, enhancing the cytotoxic effects of radiotherapy. The immune system mechanisms are also involved in the tumor suppressive properties of genistein.

This review underlined the increased therapeutic effect of radiotherapy due to genistein that inhibits DNA repair, induces cell cycle arrest, produces ROS, and modulates the apoptotic pathways in tumor cells while exerting the antioxidant, anti-inflammatory, and DNA repair effect in healthy cells. However, clinical studies on the radioprotective effect of genistein remain limited. One clinical study has demonstrated that genistein increases the effect of radiotherapy in treating pain caused by bone metastases (ClinicalTrials.gov ID NCT00769990). Another phase I clinical study determined the reduction in the side effects of radiotherapy performed by genistein on head and neck cancer patients (ClinicalTrials.gov ID NCT02075112). However, genistein has not been applied as an adjuvant radiotherapy in clinical practice. Ongoing research and clinical validation help harness the potential of genistein to improve cancer treatment outcomes. Genistein application in radiotherapy is a promising avenue to improve cancer treatment. The combination of genistein with other treatment modalities is also promising. Genistein and cisplatin have synergistic inhibitory effects on cancer cell growth [113], and genistein reduces the ototoxicity and kidney injury induced by cisplatin [114,115]. The combination of calcitriol and genistein is an attractive therapeutic option for the treatment of prostate cancer, and a genistein–topotecan combination is also an alternative adjuvant therapy for prostate cancer [116]. Tong et al. reported that genistein in combination with gefitinib inhibits hepatocellular carcinoma cell proliferation and promotes apoptosis by inhibiting the Akt/Erk/mTOR pathway [117]. Furthermore, genistein enhances the efficiency of natural killer cells in immunotherapy against cholangiocarcinoma [118]. While the prospects are encouraging, several challenges should be addressed by comprehensive research and clinical trials. Future research is necessary to integrate genistein into standard cancer treatment protocols by focusing on optimal dosing, patient variability, and long-term safety, ultimately improving patient outcomes worldwide.

The potential radioprotective effects of genistein on healthy cells and the radiosensitive effects of genistein on tumor cells increase the possibility of using it as an adjuvant in radiotherapy, reflecting the “killing two birds with one stone” approach. Additional clinical research is needed to establish the benefits by optimizing the usage of genistein in radiotherapy.

## Data Availability

Not applicable.

## References

[1] Zhou Z.H., Guan B.J., Xia H., Zheng R., Xu B.H. (2023). Particle radiotherapy in the era of radioimmunotherapy. Cancer Lett..

[2] Rosenthal A., Israilevich R., Moy R. (2019). Management of acute radiation dermatitis: A review of the literature and proposal for treatment algorithm. J. Am. Acad. Dermatol..

[3] Bower J.E. (2014). Cancer-related fatigue—Mechanisms, risk factors and treatments. Nat. Rev. Clin. Oncol..

[4] Bentzen S.M. (2006). Preventing or reducing late side effects of radiation therapy: Radiobiology meets molecular pathology. Nat. Rev. Cancer.

[5] Darby S.C., Cutter D.J., Boerma M., Constine L.S., Fajardo L.F., Kodama K., Mabuchi K., Marks L.B., Mettler F.A., Pierce L.J. (2010). Radiation-related heart disease: Current knowledge and future prospects. Int. J. Radiat. Oncol. Biol. Phys..

[6] Marks L.B., Bentzen S.M., Deasy J.O., Kong F.M., Bradley J.D., Vogelius I.S., El Naqa I., Hubbs J.L., Lebesque J.V., Timmerman R.D. (2010). Radiation dose-volume effects in the lung. Int. J. Radiat. Oncol. Biol. Phys..

[7] Ji L., Cui P., Zhou S., Qiu L., Huang H., Wang C., Wang J. (2023). Advances of Amifostine in Radiation Protection: Administration and Delivery. Mol. Pharm..

[8] Obrador E., Salvador R., Villaescusa J.I., Soriano J.M., Estrela J.M. (2020). Montoro, Radioprotection and Radiomitigation: From the Bench to Clinical Practice. Biomedicines.

[9] Henninger C., Fritz G. (2017). Statins in anthracycline-induced cardiotoxicity: Rac and Rho, and the heartbreakers. Cell Death Dis..

[10] Chung E.J., Sowers A., Thetford A., McKay-Corkum G., Chung S.I., Mitchell J.B., Citrin D.E. (2016). Mammalian Target of Rapamycin Inhibition with Rapamycin Mitigates Radiation-Induced Pulmonary Fibrosis in a Murine Model. Int. J. Radiat. Oncol. Biol. Phys..

[11] Farhood B., Goradel N.H., Mortezaee K., Khanlarkhani N., Salehi E., Nashtaei M.S., Mirtavoos-Mahyari H., Motevaseli E., Shabeeb D., Musa A.E. (2019). Melatonin as an adjuvant in radiotherapy for radioprotection and radiosensitization. Clin. Transl. Oncol..

[12] Charrier S., Michaud A., Badaoui S., Giroux S., Ezan E., Sainteny F., Corvol P., Vainchenker W. (2004). Inhibition of angiotensin I-converting enzyme induces radioprotection by preserving murine hematopoietic short-term reconstituting cells. Blood.

[13] Huang R., Zhou P.K. (2021). DNA damage repair: Historical perspectives, mechanistic pathways and clinical translation for targeted cancer therapy. Signal Transduct. Target. Ther..

[14] Liu X., Sun C., Wang Q., Li P., Zhao T., Li Q. (2023). Sp1 Upregulation Bolsters the Radioresistance of Glioblastoma Cells by Promoting Double Strand Breaks Repair. Int. J. Mol. Sci..

[15] Pawlik T.M., Keyomarsi K. (2004). Role of cell cycle in mediating sensitivity to radiotherapy. Int. J. Radiat. Oncol. Biol. Phys..

[16] Mouw J.K., Ou G., Weaver V.M. (2014). Extracellular matrix assembly: A multiscale deconstruction. Nat. Rev. Mol. Cell Biol..

[17] Olivares-Urbano M.A., Griñán-Lisón C., Marchal J.A., Núñez M.I. (2020). CSC Radioresistance: A Therapeutic Challenge to Improve Radiotherapy Effectiveness in Cancer. Cells.

[18] Perna S., Alawadhi H., Riva A., Allegrini P., Petrangolini G., Gasparri C., Alalwan T.A., Rondanelli M. (2022). In Vitro and In Vivo Anticancer Activity of Basil (*Ocimum* spp.): Current Insights and Future Prospects. Cancers.

[19] Bhat S.S., Prasad S.K., Shivamallu C., Prasad K.S., Syed A., Reddy P., Cull C.A., Amachawadi R.G. (2021). Genistein: A Potent Anti-Breast Cancer Agent. Curr. Issues Mol. Biol..

[20] Fan P., Fan S., Wang H., Mao J., Shi Y., Ibrahim M.M., Ma W., Yu X., Hou Z., Wang B. (2013). Genistein decreases the breast cancer stem-like cell population through Hedgehog pathway. Stem Cell Res. Ther..

[21] Delaney G., Jacob S., Featherstone C., Barton M. (2005). The role of radiotherapy in cancer treatment: Estimating optimal utilization from a review of evidence-based clinical guidelines. Cancer.

[22] Liu X., Li P., Hirayama R., Niu Y., Liu X., Chen W., Jin X., Zhang P., Ye F., Zhao T. (2018). Genistein sensitizes glioblastoma cells to carbon ions via inhibiting DNA-PKcs phosphorylation and subsequently repressing NHEJ and delaying HR repair pathways. Radiother. Oncol..

[23] George S., Abrahamse H. (2020). Redox Potential of Antioxidants in Cancer Progression and Prevention. Antioxidants.

[24] Najafi M., Motevaseli E., Shirazi A., Geraily G., Rezaeyan A., Norouzi F., Rezapoor S., Abdollahi H. (2018). Mechanisms of inflammatory responses to radiation and normal tissues toxicity: Clinical implications. Int. J. Radiat. Biol..

[25] Landauer M.R., Srinivasan V., Seed T.M. (2003). Genistein treatment protects mice from ionizing radiation injury. J. Appl. Toxicol..

[26] Zhou Y., Mi M.T. (2005). Genistein stimulates hematopoiesis and increases survival in irradiated mice. J. Radiat. Res..

[27] Davis T.A., Mungunsukh O., Zins S., Day R.M., Landauer M.R. (2008). Genistein induces radioprotection by hematopoietic stem cell quiescence. Int. J. Radiat. Biol..

[28] Zhang J., Pang Z., Zhang Y., Liu J., Wang Z., Xu C., He L., Li W., Zhang K., Zhang W. (2021). Genistein From *Fructus sophorae* Protects Mice From Radiation-Induced Intestinal Injury. Front. Pharmacol..

[29] Son T.G., Gong E.J., Bae M.J., Kim S.D., Heo K., Moon C., Yang K., Kim J.S. (2013). Protective effect of genistein on radiation-induced intestinal injury in tumor bearing mice. BMC Complement. Altern. Med..

[30] Day R.M., Barshishat-Kupper M., Mog S.R., McCart E.A., Prasanna P.G., Davis T.A., Landauer M.R. (2008). Genistein protects against biomarkers of delayed lung sequelae in mice surviving high-dose total body irradiation. J. Radiat. Res..

[31] Calveley V.L., Jelveh S., Langan A., Mahmood J., Yeung I.W., Van Dyk J., Hill R.P. (2010). Genistein can mitigate the effect of radiation on rat lung tissue. Radiat. Res..

[32] Uslu G.H., Canyilmaz E., Serdar L., Ersöz Ş. (2019). Protective effects of genistein and melatonin on mouse liver injury induced by whole-body ionising radiation. Mol. Clin. Oncol..

[33] Kim J.S., Heo K., Yi J.M., Gong E.J., Yang K., Moon C., Kim S.H. (2012). Genistein mitigates radiation-induced testicular injury. Phytother. Res..

[34] Jackson I.L., Pavlovic R., Alexander A.A., Connors C.Q., Newman D., Mahmood J., Eley J., Harvey A.J., Kaytor M.D., Vujaskovic Z. (2019). BIO 300, a Nanosuspension of Genistein, Mitigates Radiation-Induced Erectile Dysfunction and Sensitizes Human Prostate Cancer Xenografts to Radiation Therapy. Int. J. Radiat. Oncol. Biol. Phys..

[35] Ahmad I.U., Forman J.D., Sarkar F.H., Hillman G.G., Heath E., Vaishampayan U., Cher M.L., Andic F., Rossi P.J., Kucuk O. (2010). Soy isoflavones in conjunction with radiation therapy in patients with prostate cancer. Nutr. Cancer.

[36] Hillman G.G., Singh-Gupta V., Runyan L., Yunker C.K., Rakowski J.T., Sarkar F.H., Miller S., Gadgeel S.M., Sethi S., Joiner M.C. (2011). Soy isoflavones radiosensitize lung cancer while mitigating normal tissue injury. Radiother. Oncol..

[37] Zhuang X.L., Fu Y.C., Xu J.J., Kong X.X., Chen Z.G., Luo L.L. (2010). Effects of genistein on ovarian follicular development and ovarian life span in rats. Fitoterapia.

[38] Goh Y.X., Jalil J., Lam K.W., Husain K., Premakumar C.M. (2022). Genistein: A Review on its Anti-Inflammatory Properties. Front. Pharmacol..

[39] Speroni F., Rebolledo A., Salemme S., Roldán-Palomo R., Rimorini L., Añón M.C., Spinillo A., Tanzi F., Milesi V. (2009). Genistein effects on Ca^2+^ handling in human umbilical artery: Inhibition of sarcoplasmic reticulum Ca^2+^ release and of voltage-operated Ca^2+^ channels. J. Physiol. Biochem..

[40] Speroni F., Rebolledo A., Salemme S., Añón M.C., Tanzi F., Milesi V. (2007). Genistein inhibits contractile force, intracellular Ca^2+^ increase and Ca^2+^ oscillations induced by serotonin in rat aortic smooth muscle. J. Physiol. Biochem..

[41] Knudson C.M., Brown N.M. (2008). Mitochondria potential; bax “activation,” and programmed cell death. Methods Mol. Biol..

[42] Wang T.S., Coppens I., Saorin A., Brady N.R., Hamacher-Brady A. (2020). Endolysosomal Targeting of Mitochondria Is Integral to BAX-Mediated Mitochondrial Permeabilization during Apoptosis Signaling. Dev. Cell.

[43] Haddad Y.H., Said R.S., Kamel R., Morsy E.M.E., El-Demerdash E. (2020). Phytoestrogen genistein hinders ovarian oxidative damage and apoptotic cell death-induced by ionizing radiation: Co-operative role of ER-β, TGF-β, and FOXL-2. Sci. Rep..

[44] Song L., Ma L., Cong F., Shen X., Jing P., Ying X., Zhou H., Jiang J., Fu Y., Yan H. (2015). Radioprotective effects of genistein on HL-7702 cells via the inhibition of apoptosis and DNA damage. Cancer Lett..

[45] Kruk I., Aboul-Enein H.Y., Michalska T., Lichszteld K., Kładna A. (2005). Scavenging of reactive oxygen species by the plant phenols genistein and oleuropein. Luminescence.

[46] Yen G.C., Lai H.H. (2003). Inhibition of reactive nitrogen species effects in vitro and in vivo by isoflavones and soy-based food extracts. J. Agric. Food Chem..

[47] Si H., Liu D. (2007). Phytochemical genistein in the regulation of vascular function: New insights. Curr. Med. Chem..

[48] Sharifi-Rad J., Quispe C., Imran M., Rauf A., Nadeem M., Gondal T.A., Ahmad B., Atif M., Mubarak M.S., Sytar O. (2021). Genistein: An Integrative Overview of Its Mode of Action, Pharmacological Properties, and Health Benefits. Oxid. Med. Cell Longev..

[49] Liu G.D., Xia L., Zhu J.W., Ou S., Li M.X., He Y., Luo W., Li J., Zhou Q., Yang X.Q. (2014). Genistein alleviates radiation-induced pneumonitis by depressing Ape1/Ref-1 expression to down-regulate inflammatory cytokines. Cell Biochem. Biophys..

[50] Mazumder M.A.R., Hongsprabhas P. (2016). Genistein as antioxidant and antibrowning agents in in vivo and in vitro: A review. Biomed. Pharmacother..

[51] Luo M., Yang Z.Q., Huang J.C., Wang Y.S., Guo B., Yue Z.P. (2020). Genistein protects ovarian granulosa cells from oxidative stress via cAMP-PKA signaling. Cell Biol. Int..

[52] Shirvanian K., Vali R., Farkhondeh T., Abderam A., Aschner M., Samarghandian S. (2024). Genistein Effects on Various Human Disorders Mediated via Nrf2 Signaling. Curr. Mol. Med..

[53] Wang K., Hu S., Wang B., Wang J., Wang X., Xu C. (2019). Genistein protects intervertebral discs from degeneration via Nrf2-mediated antioxidant defense system: An in vitro and in vivo study. J. Cell. Physiol..

[54] Li Y., Zhang J.J., Chen R.J., Chen L., Chen S., Yang X.F., Min J.W. (2022). Genistein mitigates oxidative stress and inflammation by regulating Nrf2/HO-1 and NF-κB signaling pathways in hypoxic-ischemic brain damage in neonatal mice. Ann. Transl. Med..

[55] Liu X.X., Sun C., Liu B.T., Jin X.D., Li P., Zheng X.G., Zhao T., Li F.F., Li Q. (2016). Genistein mediates the selective radiosensitizing effect in NSCLC A549 cells via inhibiting methylation of the keap1 gene promoter region. Oncotarget.

[56] Shi B.W., He E.Y., Chang K.L., Xu G.D., Meng Q.Y., Xu H.H., Chen Z.Y., Wang X.J., Jia M., Sun W. (2024). Genistein prevents the production of hypospadias induced by Di-(2-ethylhexyl) phthalate through androgen signaling and antioxidant response in rats. J. Hazard Mater..

[57] Lu H., Shi J.X., Zhang D.M., Wang H.D., Hang C.H., Chen H.L., Yin H.X. (2009). Inhibition of hemolysate-induced iNOS and COX-2 expression by genistein through suppression of NF-small ka, CyrillicB activation in primary astrocytes. J. Neurol. Sci..

[58] Terra V.A., Souza-Neto F.P., Frade M.A., Ramalho L.N., Andrade T.A., Pasta A.A., Conchon A.C., Guedes F.A., Luiz R.C., Cecchini R. (2015). Genistein prevents ultraviolet B radiation-induced nitrosative skin injury and promotes cell proliferation. J. Photochem. Photobiol. B.

[59] Liu T., Zhang L., Joo D., Sun S.C. (2017). NF-κB Signaling in Inflammation. Signal Transduct. Target. Ther..

[60] Xie X., Cong L., Liu S., Xiang L., Fu X. (2021). Genistein alleviates chronic vascular inflammatory response via the miR-21/NF-κB p65 axis in lipopolysaccharide-treated mice. Mol. Med. Rep..

[61] Ji G., Zhang Y., Yang Q., Cheng S., Hao J., Zhao X., Jiang Z. (2012). Genistein Suppresses LPS-Induced Inflammatory Response through Inhibiting NF-Κb Following AMP Kinase Activation in RAW 264.7 Macrophages. PLoS ONE.

[62] Zhou X., Yuan L., Zhao X., Hou C., Ma W., Yu H., Xiao R. (2014). Genistein Antagonizes Inflammatory Damage Induced by β-amyloid Peptide in Microglia through TLR4 and NF-κb. Nutrition.

[63] Raffoul J.J., Wang Y., Kucuk O., Forman J.D., Sarkar F.H., Hillman G.G. (2006). Genistein inhibits radiation-induced activation of NF-kappaB in prostate cancer cells promoting apoptosis and G_2_/M cell cycle arrest. BMC Cancer.

[64] Haddad Y. (2019). Genistein as Radioprotective Against Premature Ovarian Failure. Al-Azhar J. Pharm. Sci..

[65] Fischer N., Seo E.J., Efferth T. (2018). Prevention from radiation damage by natural products. Phytomedicine.

[66] Kim D.H., Jung W.S., Kim M.E., Lee H.W., Youn H.Y., Seon J.K., Lee H.N., Lee J.S. (2014). Genistein inhibits pro-inflammatory cytokines in human mast cell activation through the inhibition of the ERK pathway. Int. J. Mol. Med..

[67] Ha C.T., Li X.H., Fu D., Xiao M., Landauer M.R. (2013). Genistein nanoparticles protect mouse hematopoietic system and prevent proinflammatory factors after gamma irradiation. Radiat. Res..

[68] Para A.E., Bezjak A., Yeung I.W., Van Dyk J., Hill R.P. (2009). Effects of genistein following fractionated lung irradiation in mice. Radiother. Oncol..

[69] Abernathy L.M., Fountain M.D., Rothstein S.E., David J.M., Yunker C.K., Rakowski J., Lonardo F., Joiner M.C., Hillman G.G. (2015). Soy Isoflavones Promote Radioprotection of Normal Lung Tissue by Inhibition of Radiation-Induced Activation of Macrophages and Neutrophils. J. Thorac. Oncol..

[70] Fountain M.D., McLellan L.A., Smith N.L., Loughery B.F., Rakowski J.T., Tse H.Y., Hillman G.G. (2020). Isoflavone-mediated radioprotection involves regulation of early endothelial cell death and inflammatory signaling in Radiation-Induced lung injury. Int. J. Radiat. Biol..

[71] Alfonso J.C.L., Berk L. (2019). Modeling the effect of intratumoral heterogeneity of radiosensitivity on tumor response over the course of fractionated radiation therapy. Radiat. Oncol..

[72] Santivasi W.L., Xia F. (2014). Ionizing radiation-induced DNA damage, response, and repair. Antioxid. Redox Signal..

[73] Zhou Y., Paull T.T. (2013). DNA-dependent protein kinase regulates DNA end resection in concert with Mre11-Rad50-Nbs1 (MRN) and ataxia telangiectasia-mutated (ATM). J. Biol. Chem..

[74] Abbasi S., Parmar G., Kelly R.D., Balasuriya N., Schild-Poulter C. (2021). The Ku complex: Recent advances and emerging roles outside of non-homologous end-joining. Cell. Mol. Life Sci..

[75] Mao Z., Bozzella M., Seluanov A., Gorbunova V. (2008). Comparison of nonhomologous end joining and homologous recombination in human cells. DNA Repair.

[76] Anderson J.A., Harper J.V., Cucinotta F.A., O’Neill P. (2010). Participation of DNA-PKcs in DSB repair after exposure to high- and low-LET radiation. Radiat. Res..

[77] Tang Q., Ma J., Sun J., Yang L., Yang F., Zhang W., Li R., Wang L., Wang Y., Wang H. (2018). Genistein1024 synergistically increase the radiosensitivity of prostate cancer cells. Oncol. Rep..

[78] Singh-Gupta V., Joiner M.C., Runyan L., Yunker C.K., Sarkar F.H., Miller S., Gadgeel S.M., Konski A.A., Hillman G.G. (2011). Soy isoflavones augment radiation effect by inhibiting APE1/Ref-1 DNA repair activity in non-small cell lung cancer. J. Thorac. Oncol..

[79] Park C., Cha H.J., Lee H., Hwang-Bo H., Ji S.Y., Kim M.Y., Hong S.H., Jeong J.W., Han M.H., Choi S.H. (2019). Induction of G_2_/M Cell Cycle Arrest and Apoptosis by Genistein in Human Bladder Cancer T24 Cells through Inhibition of the ROS-Dependent PI3k/Akt Signal Transduction Pathway. Antioxidants.

[80] Ouyang G., Yao L., Ruan K., Song G., Mao Y., Bao S. (2009). Genistein induces G_2_/M cell cycle arrest and apoptosis of human ovarian cancer cells via activation of DNA damage checkpoint pathways. Cell Biol. Int..

[81] Dillon M.T., Good J.S., Harrington K.J. (2014). Selective targeting of the G_2_/M cell cycle checkpoint to improve the therapeutic index of radiotherapy. Clin. Oncol..

[82] Liu C., Nie J., Wang R.S., Mao W.D. (2019). The Cell Cycle G_2_/M Block Is an Indicator of Cellular Radiosensitivity. Dose Response.

[83] Chang K.L., Kung M.L., Chow N.H., Su S.J. (2004). Genistein arrests hepatoma cells at G_2_/M phase: Involvement of ATM activation and upregulation of p21waf1/cip1 and Wee1. Biochem. Pharmacol..

[84] Zhang Z., Wang C.Z., Du G.J., Qi L.W., Calway T., He T.C., Du W., Yuan C.S. (2013). Genistein induces G_2_/M cell cycle arrest and apoptosis via ATM/p53-dependent pathway in human colon cancer cells. Int. J. Oncol..

[85] Liu X., Sun C., Jin X., Li P., Ye F., Zhao T., Gong L., Li Q. (2013). Genistein enhances the radiosensitivity of breast cancer cells via G_2_/M cell cycle arrest and apoptosis. Molecules.

[86] Yan H., Jiang J., Du A., Gao J., Zhang D., Song L. (2020). Genistein Enhances Radiosensitivity of Human Hepatocellular Carcinoma Cells by Inducing G_2_/M Arrest and Apoptosis. Radiat. Res..

[87] Zhang B., Liu J.Y., Pan J.S., Han S.P., Yin X.X., Wang B., Hu G. (2006). Combined treatment of ionizing radiation with genistein on cervical cancer HeLa cells. J. Pharmacol. Sci..

[88] Spagnuolo C., Russo G.L., Orhan I.E., Habtemariam S., Daglia M., Sureda A., Nabavi S.F., Devi K.P., Loizzo M.R., Tundis R. (2015). Genistein and cancer: Current status, challenges, and future directions. Adv. Nutr..

[89] Kim I.G., Kim J.S., Lee J.H., Cho E.W. (2014). Genistein decreases cellular redox potential, partially suppresses cell growth in HL-60 leukemia cells and sensitizes cells to γ-radiation-induced cell death. Mol. Med. Rep..

[90] Hunter A.J., Hendrikse A.S., Renan M.J. (2009). Radiation-induced apoptosis is modulated by the post-irradiation tumor microenvironment. Cancer Biol. Ther..

[91] De Paula M.L., Rodrigues D.H., Teixeira H.C., Barsante M.M., Souza M.A., Ferreira A.P. (2008). Genistein down-modulates pro-inflammatory cytokines and reverses clinical signs of experimental autoimmune encephalomyelitis. Int. Immunopharmacol..

[92] Liu X., Liu K., Qin J., Hao L., Li X., Liu Y., Zhang X., Liu X., Li P., Han S. (2015). C/EBPβ promotes angiogenesis through secretion of IL-6, which is inhibited by genistein, in EGFRvIII-positive glioblastoma. Int. J. Cancer.

[93] Sekar V., Anandasadagopan S.K., Ganapasam S. (2016). Genistein regulates tumor microenvironment and exhibits anticancer effect in dimethyl hydrazine-induced experimental colon carcinogenesis. Biofactors.

[94] Monjazeb A.M., Schalper K.A., Villarroel-Espindola F., Nguyen A., Shiao S.L., Young K. (2020). Effects of Radiation on the Tumor Microenvironment. Semin. Radiat. Oncol..

[95] Erten F., Yenice E., Orhan C., Er B., Öner P.D., Deeh P.B.D., Şahin K. (2021). Genistein suppresses the inflammation and GSK-3 pathway in an animal model of spontaneous ovarian cancer. Turk. J. Med. Sci..

[96] Al-Khayri J.M., Sahana G.R., Nagella P., Joseph B.V., Alessa F.M., Al-Mssallem M.Q. (2022). Flavonoids as Potential Anti-Inflammatory Molecules: A Review. Molecules.

[97] Hermann R.M., Wolff H.A., Jarry H., Thelen P., Gruendker C., Rave-Fraenk M., Schmidberger H., Christiansen H. (2008). In vitro studies on the modification of low-dose hyper-radiosensitivity in prostate cancer cells by incubation with genistein and estradiol. Radiat. Oncol..

[98] Akimoto T., Nonaka T., Ishikawa H., Sakurai H., Saitoh J.I., Takahashi T., Mitsuhashi N. (2001). Genistein, a tyrosine kinase inhibitor, enhanced radiosensitivity in human esophageal cancer cell lines in vitro: Possible involvement of inhibition of survival signal transduction pathways. Int. J. Radiat. Oncol. Biol. Phys..

[99] Das A., Banik N.L., Ray S.K. (2010). Flavonoids activated caspases for apoptosis in human glioblastoma T98G and U87MG cells but not in human normal astrocytes. Cancer.

[100] Kaytor M.D., Serebrenik A.A., Lapanowski K., McFall D., Jones M., Movsas B., Simone C.B., Brown S.L. (2023). The radioprotectant nano-genistein enhances radiotherapy efficacy of lung tumors in mice. Transl. Lung Cancer Res..

[101] Hillman G.G. (2019). Soy Isoflavones Protect Normal Tissues While Enhancing Radiation Responses. Semin. Radiat. Oncol..

[102] Hillman G.G., Singh-Gupta V., Hoogstra D.J., Abernathy L., Rakowski J., Yunker C.K., Rothstein S.E., Sarkar F.H., Gadgeel S., Konski A.A. (2013). Differential effect of soy isoflavones in enhancing high intensity radiotherapy and protecting lung tissue in a pre-clinical model of lung carcinoma. Radiother. Oncol..

[103] Akbari S., Kariznavi E., Jannati M., Elyasi S., Tayarani-Najaran Z. (2020). Curcumin as a preventive or therapeutic measure for chemotherapyand radiotherapy induced adverse reaction: A comprehensive review. Food Chem. Toxicol..

[104] Boretti A. (2024). Evidence for the use of curcumin in radioprotection and radiosensitization. Phytother. Res..

[105] Sebastià N., Montoro A., Hervás D., Pantelias G., Hatzi V.I., Soriano J.M., Villaescusa J.I., Terzoudi G.I. (2014). Curcumin and trans-resveratrol exert cell cycle-dependent radioprotective or radiosensitizing effects as elucidated by the PCC and G2-assay. Mutat. Res..

[106] Komorowska D., Radzik T., Kalenik S., Rodacka A. (2022). Natural Radiosensitizers in Radiotherapy: Cancer Treatment by Combining Ionizing Radiation with Resveratrol. Int. J. Mol. Sci..

[107] Zhang Y.M., Miao Z.M., Chen Y.P., Song Z.B., Li Y.Y., Liu Z.W., Zhou G.C., Li J., Shi L.L., Chen Y. (2024). Ononin promotes radiosensitivity in lung cancer by inhibiting HIF-1α/VEGF pathway. Phytomedicine.

[108] Jeong J.W., Lee H.H., Han M.H., Kim G.Y., Kim W.J., Choi Y.H. (2014). Anti-inflammatory effects of genistein via suppression of the toll-like receptor 4-mediated signaling pathway in lipopolysaccharide-stimulated BV2 microglia. Chem. Biol. Interact..

[109] Yoon G.A., Park S. (2014). Antioxidant Action of Soy Isoflavones on Oxidative Stress and Antioxidant Enzyme Activities in Exercised Rats. Nutr. Res. Pract..

[110] Cheng W.X., Huang H., Chen J.H., Zhang T.T., Zhu G.Y., Zheng Z.T., Lin J.T., Hu Y.P., Zhang Y., Bai X.L. (2019). Genistein inhibits angiogenesis developed during rheumatoid arthritis through the IL-6/JAK2/STAT3/VEGF signalling pathway. J. Orthop. Translat..

[111] Ardito F., Di Gioia G., Pellegrino M.R., Muzio L.L. (2018). Genistein as a Potential Anticancer Agent Against Head and Neck Squamous Cell Carcinoma. Curr. Top. Med. Chem..

[112] Hagiwara H., Wako H., Nakata K., Aida R. (2023). Genistein Induces Antiproliferative Activity and Apoptosis in Human Osteosarcoma Saos-2 Cells. Anticancer. Res..

[113] Khoshyomn S., Manske G.C., Lew S.M., Wald S.L., Penar P.L. (2000). Synergistic action of genistein and cisplatin on growth inhibition and cytotoxicity of human medulloblastoma cells. Pediatr. Neurosurg..

[114] Tan M., Toplu Y., Varan E., Sapmaz E., Özhan O., Parlakpınar H., Polat A. (2022). The effect of genistein on cisplatin induced ototoxicity and oxidative stress. Braz. J. Otorhinolaryngol..

[115] Sung M.J., Kim D.H., Jung Y.J., Kang K.P., Lee A.S., Lee S., Kim W., Davaatseren M., Hwang J.T., Kim H.J. (2008). Genistein protects the kidney from cisplatin-induced injury. Kidney Int..

[116] Swami S., Krishnan A.V., Moreno J., Bhattacharyya R.B., Peehl D.M., Feldman D. (2007). Calcitriol and genistein actions to inhibit the prostaglandin pathway: Potential combination therapy to treat prostate cancer. J. Nutr..

[117] Hörmann V., Kumi-Diaka J., Durity M., Rathinavelu A. (2012). Anticancer activities of genistein-topotecan combination in prostate cancer cells. J. Cell Mol. Med..

[118] Tong Y., Wang M., Huang H., Zhang J., Huang Y., Chen Y., Pan H. (2019). Inhibitory effects of genistein in combination with gefitinib on the hepatocellular carcinoma Hep3B cell line. Exp. Ther. Med..

